# Transmission Electron Microscopy to Follow Ultrastructural Modifications of Erythroblasts Upon *ex vivo* Human Erythropoiesis

**DOI:** 10.3389/fphys.2021.791691

**Published:** 2022-02-09

**Authors:** Alice Dussouchaud, Julieta Jacob, Charles Secq, Jean-Marc Verbavatz, Martina Moras, Jérôme Larghero, Claudio M. Fader, Mariano A. Ostuni, Sophie D. Lefevre

**Affiliations:** ^1^Université de Paris and Université des Antilles, INSERM, BIGR, Paris, France; ^2^Laboratorio de Biología Celular y Molecular, Instituto de Histología y Embriología, CONICET, Universidad Nacional de Cuyo, Mendoza, Argentina; ^3^Facultad de Odontología, Universidad Nacional de Cuyo, Mendoza, Argentina; ^4^CNRS, UMR 7592, Institut Jacques Monod, Université de Paris, Paris, France

**Keywords:** erythropoiesis, electron microscopy, mitochondria, autophagy, vesicles

## Abstract

Throughout mammal erythroid differentiation, erythroblasts undergo enucleation and organelle clearance becoming mature red blood cell. Organelles are cleared by autophagic pathways non-specifically targeting organelles and cytosolic content or by specific mitophagy targeting mitochondria. Mitochondrial functions are essential to coordinate metabolism reprogramming, cell death, and differentiation balance, and also synthesis of heme, the prosthetic group needed in hemoglobin assembly. In mammals, mitochondria subcellular localization and mitochondria interaction with other structures as endoplasmic reticulum and nucleus might be of importance for the removal of the nucleus, that is, the enucleation. Here, we aim to characterize by electron microscopy the changes in ultrastructure of cells over successive stages of human erythroblast differentiation. We focus on mitochondria to gain insights into intracellular localization, ultrastructure, and contact with other organelles. We found that mitochondria are progressively cleared with a significant switch between PolyE and OrthoE stages, acquiring a rounded shape and losing contact sites with both ER (MAM) and nucleus (NAM). We studied intracellular vesicle trafficking and found that endosomes and MVBs, known to be involved in iron traffic and heme synthesis, are increased during BasoE to PolyE transition; autophagic structures such as autophagosomes increase from ProE to OrthoE stages. Finally, consistent with metabolic switch, glycogen accumulation was observed in OrthoE stage.

## Introduction

Erythropoiesis is a finely regulated process producing two million erythrocytes every second in healthy human adults (Palis, 2014). During the late phase of erythropoiesis, called terminal erythropoiesis, proerythroblast (ProE) undergoes several cellular divisions to give rise to basophilic (BasoE), polychromatic (PolyE), and orthochromatic (OrthoE) erythroblasts, successively. Through these divisions, morphological changes, such as cell size reduction and chromatin condensation, will occur (Wickramasinghe et al., 2011). The nomenclature of these stages is given by morphological description after May-Grünwald-Giemsa coloration (Wickramasinghe et al., 2011). Indeed, the ProE has a large nucleus and the cytoplasm contain free ribosomes giving it a basophilic (purple) appearance. The BasoE is smaller, which has a more basophilic cytoplasm due to increased numbers of ribosomes and chromatin condenses. The PolyE appears grayer due to the increased acidophilic staining caused by the presence of hemoglobin. Cell division stops at this stage. At the end, the OrthoE has more acidophilic (pink) appearance. At this stage, the transcription stops and the nucleus becomes pyknotic, as the chromatin condenses, and polarizes to prepare for enucleation (Wickramasinghe et al., 1968, 2011). At the end of human terminal erythropoiesis, OrthoE gave rise to reticulocyte, a cell devoid of nucleus, which migrates toward the blood stream and matures into red blood cell.

During this last phase of erythropoiesis, some specific proteins, such as hemoglobin or the membrane protein Band 3, will be expressed under the control of specific transcription factors to allow the cell to become highly specialized (Peters et al., 1992; Liu et al., 2011; Fan et al., 2015). Large amount of iron will be imported in the first stages of terminal erythropoiesis to fulfill the cell requirement for heme synthesis (Fontenay et al., 2006; Dailey and Meissner, 2013). In parallel, some other proteins will be specifically removed (Blanc et al., 2005; Gautier et al., 2016). In addition, organelles including the Golgi apparatus, endoplasmic reticulum (ER), ribosomes, and mitochondria are eliminated (Moras et al., 2017). Organelles can be eliminated by the general process of macroautophagy, which starts by the formation of a double membrane structure called phagophore, engulfing cytoplasmic material to form the autophagosome which fuses with a lysosome for degradation (Klionsky, 2005). All these processes including membrane remodeling, changes in metabolic pathways, and organelle clearance involved high intracellular vesicle trafficking such as the generation and dynamic of endosomes, multivesicular bodies, or autophagosomes.

Mitochondria can specifically be removed by mitophagy (Sandoval et al., 2008; Moras et al., 2020, 2022). A prerequisite for mitochondrial clearance is mitochondria fragmentation, as impaired fragmentation results in the inhibition hemoglobin biosynthesis and erythropoiesis (Gonzalez-Ibanez et al., 2020). Mitochondria are also known to form contact sites with other organelles such as ER, lipid droplets, endosomes, lysosomes, plasma membranes, and nucleus (Lackner, 2019). These membrane-to-membrane contact sites are essential to exchange metabolites and to regulate several pathways allowing cellular adaptation to metabolic changes and cellular stress (Giamogante et al., 2020). Even so the mitochondria contact sites are not well studied throughout erythropoiesis, a recent elegant article demonstrated that a perinuclear mitochondria localization is necessary to achieve the enucleation of orthochromatic erythroblasts in mice (Liang et al., 2021). Furthermore, we have recently described a reduction of mitochondria to ER contact site number in a context of impaired terminal erythroid maturation (Moras et al., 2022).

Only a few very interesting papers have partially described ultrastructural changes during erythropoiesis in mammals (Betin et al., 2013; Gonzalez-Ibanez et al., 2020; Moras et al., 2022). The more exhaustive characterization of the progressive changes in endomembrane compartments during human erythroid differentiation was reported by Betin et al. (2013) who described the appearance of different autophagic compartments and the reduction in the number of mitochondria.

Mitochondrial retention was previously associated with deficient mitophagy following mitophagy receptors knockdown in mice (Sandoval et al., 2008) and humans (Moras et al., 2022). During the last 5 years, growing evidences were reported correlating autophagy deficiencies and particularly mitophagy defaults with different pathologies as sickle cell disease (SCD) or systemic lupus erythematosus (SLE; Jagadeeswaran et al., 2017; Caielli et al., 2021; Martino et al., 2021). Moreover, a novel atypical anemia was associated with the presence of miscellaneous membranous structure on patients’ reticulocytes and mature red blood (Ru et al., 2018). Then, the whole characterization of ultrastructural changes during erythroid terminal differentiation is a necessary tool helping to analyze and understand pathological anomalies specially found in dyserythropoiesis.

Herein, we performed human *ex vivo* erythropoiesis and sorted different erythroblasts stages during terminal erythropoiesis to identify and quantify by electron microscopy the ultrastructural features appearing throughout differentiation steps. We characterized cell shape, nucleus localization, chromatin condensation, mitochondrial distribution and shape, and also the presence of mitochondrial-ER and mitochondrial nucleus contact site. We also quantified endomembrane vesicles such as multivesicular bodies, endosomes, and autophagosomes through differentiation stages. Finally, we identified the appearance of glycogen stores at the late stages of differentiation, as a signal of metabolic switch.

## Materials and Methods

### Human CD34-Positive Cell Purification and Differentiation

Cord bloods from healthy donors from Saint-Louis Hospital Cord Blood Bank registered to the French Ministry of Research under number AC-2016-2756 and to the French Normalization Agency under number 201/51848.1 were used in this study. This study was approved and conducted according to Institutional Ethical Guidelines of the National Institute of Blood Transfusion (N°2019-1, INTS, Paris, France). All procedures were carried out in accordance with the Declaration of Helsinki. Written informed consent was given by the donors. Mononuclear cells were separated from blood using Ficoll-Paque (GE Healthcare), and CD34^+^ cells were enriched using magnetic cell sorting beads (Miltenyi Biotec, Paris, France) according to the manufacturer’s instruction.

**FIGURE 1:**
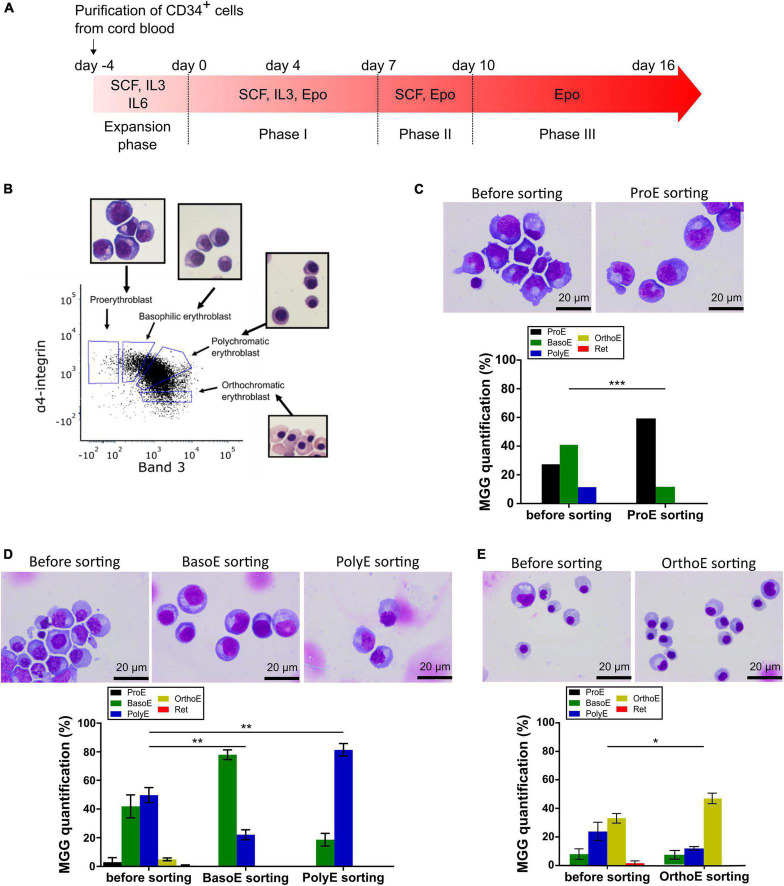
Erythroblast enrichment prior analysis by electron microscopy. **(A)** Time course of CD34-positive cells *ex vivo* differentiation with the list of used cytokines at each stage. **(B)** Validation of sorting gates by MGG analysis of sorted cells on a representative flow cytometry panel of GPA-positive cells gated according to α-integrin and Band 3 protein expression at day 10 of differentiation. **(C–E)** MGG representative pictures (top) and quantification (bottom) of erythroid cells before and after sorting at day 6 **(A)**, day 12 **(B)**, and day 15 **(C)**. Mann–Whitney test was used. *p < 0.05; **p < 0.01; ***p < 0.005.

CD34^+^ cells were cultured in Iscove’s Modified Dulbecco’s Medium (IMDM) GlutaMAX™ (Thermo Fischer Scientific, Courtaboeuf, France), 2% human peripheral blood plasma (STEMCELL Technologies, Saint-Égrève, France), 3% human AB serum (Merck KGaA, Darmstadt, Germany), 15% BIT 9500 serum substitute (STEMCELL Technologies), and 3 IU/mL heparin (Merck KGaA). During expansion phase (day −4 to day 0), cells were seeded at a concentration of 10^5^ cells/mL in culture medium supplemented with 25 ng/mL stem cell factor (hSCF), 10 ng/mL IL-3, 10 ng/mL IL-6 from Miltenyi Biotec. From day 0 to day 4, medium was supplemented with 10 μg/mL insulin (Merck, KGaA), 200 μg/mL human holo-transferrin (Merck KGaA), 10% penicillin/streptomycin, 10 ng/mL SCF, 1 ng/mL IL-3, and 3 IU/mL erythropoietin (EPO), whereas IL-6 and BIT were removed from the medium. From day 7, IL-3 was omitted from culture media and from day 10, and hSCF was removed. Holo-transferrin concentration was increased up to 1,000 μg/mL. Cell concentration was maintained between 10^5^ and 10^6^ at 37°C with the presence of 5% CO_2_. Figure 1A presents the culture protocol.

### Flow Cytometry-Based Erythroid Differentiation Analysis and Erythroblast Sorting

ProE, BasoE, and PolyE, and OrthoE were sorted at day 6, 12, and 15, respectively, based on the expression of surface markers expression as described by Hu et al. (2013). Briefly, 20 million cells were incubated with PBS 0.5% BSA supplemented with 10% AB serum for 10 min at 4°C for blocking. BV510-coupled antiglycophorin A (GPA) antibody (1/25e), APC-Cy7-coupled anti-α4-integrin antibody (1/8e), and PE-coupled anti-band 3 antibody (1/16e) were added to the cells. After 20 min of incubation at 4°C, cells were washed with PBS 0.5% BSA, and 7-amino-actinomycin-D (7-AAD, BD Biosciences) (1/200e) was added just before reading to exclude dead cells. For orthochromatic erythroblast sorting, 1 μg/mL Hoechst 34580 (BD Biosciences) was added to the culture and incubated 30 min at 37°C prior to antibodies labeling. Cells were sorted using the cell sorter SONY MA900.

### May-Grünwald Giemsa-Based Identification of Erythroblasts

Morphological quantifications were performed on 10^5^ cells, before and after sorting, cytospin on a glass slide for 3 min at 300 G using the Thermo Fisher Shandon 4 Cytospin. After drying, slides were stained with 5 min in May-Grünwald (MG) solution (Merck KGaA), transferred in 1/2-diluted MG solution for 5 min, washed in potassium phosphate buffer 8.5 mM pH 7.2 for 3 min, incubated in 1/20-diluted Giemsa solution (Merck KGaA) for 15 min, and washed in phosphate buffer 8.5 mM pH 7.2 for 3 min before drying. Cells were then imaged using Leica DMI4000 microscope 100x/0.6 objective. A number of 30–35 cells per sample were analyzed.

### Cell Preparation for Electron Microscopy and Image Acquisition

After sorting, 3–5 million cells were immediately fixed with 1% glutaraldehyde/2.5% paraformaldehyde for at least 2 h. After washing, cells were postfixed with 1% osmium tetroxide reduced with 1.5% potassium ferrocyanide in PBS (pH 7.4), progressively dehydrated in ethanol, and embedded in low-viscosity epoxy resin. However, 70-nm-thin sections were cut, mounted on copper grids, and stained with uranyl acetate and lead citrate. Sections were examined with a 120 kV TEM (Tecnai 12, Thermo Fischer Scientific) equipped with a 4K CDD camera (OneView, Gatan). At least 20 images per replicates were analyzed using 3DMOD and IMOD or FIJI software depending on the application.

### Statistical Tests

Erythroid differentiation was repeated in triplicates, with a satisfactory correlation between the results of individual experiments. Statistical analyses were performed using Prism 8 (GraphPad Software). Data were evaluated using Mann–Whitney or Kruskal–Wallis tests, and all comparisons with a *p*-value less than 0.05 were considered statistically significant.

## Results

### Electron Microscopy to Identify Erythroblasts

Relatively recent works characterized membrane protein expression in erythroblast stages, which generate a standardized protocol for human erythroblast identification and sorting by flow cytometry (Chen et al., 2009; Hu et al., 2013). To generate from ProE to OrthoE, differentiation of cord blood purified CD34-positive cells was started by adding EPO (day 0). The expression of α4-integrin and Band 3 on glycophorin (GPA)-positive cells was analyzed by flow cytometry to quantify the proportion of ProE, BasoE, PolyE, and OrthoE. Cells were sorted accordingly, May-Grünwald Giemsa (MGG) staining was used to validate the gating on a standard experiment, and gates were not changed afterward (Figure 1B).

As differentiation progresses, various days after EPO addition were selected to sorted different erythroblast stages (Figure 1A). With 27.37% of ProE prior sorting, day 6 after EPO addition was used to sort this stage; BasoE and PolyE were the most frequent stages at day 12 after EPO addition, and 35 ± 5.7% of cells were OrthoE at day 15 after EPO addition. The enrichment of sorted fractions was evaluated based on MGG staining by calculating the ratio expressed in percent of the representativity of each stage after related to before sorting. For ProE fractions, the enrichment was 310% for each replicate (Figure 1C). For BasoE and PolyE, it was 202, 146, and 224% and 143, 196, and 156%, respectively (Figure 1D). For OrthoE fractions, the enrichment ratio is of 176, 129, and 132% for the tree replicates (Figure 1E). For all stages, the enrichment was statistically significant (Figure 1).

**FIGURE 2:**
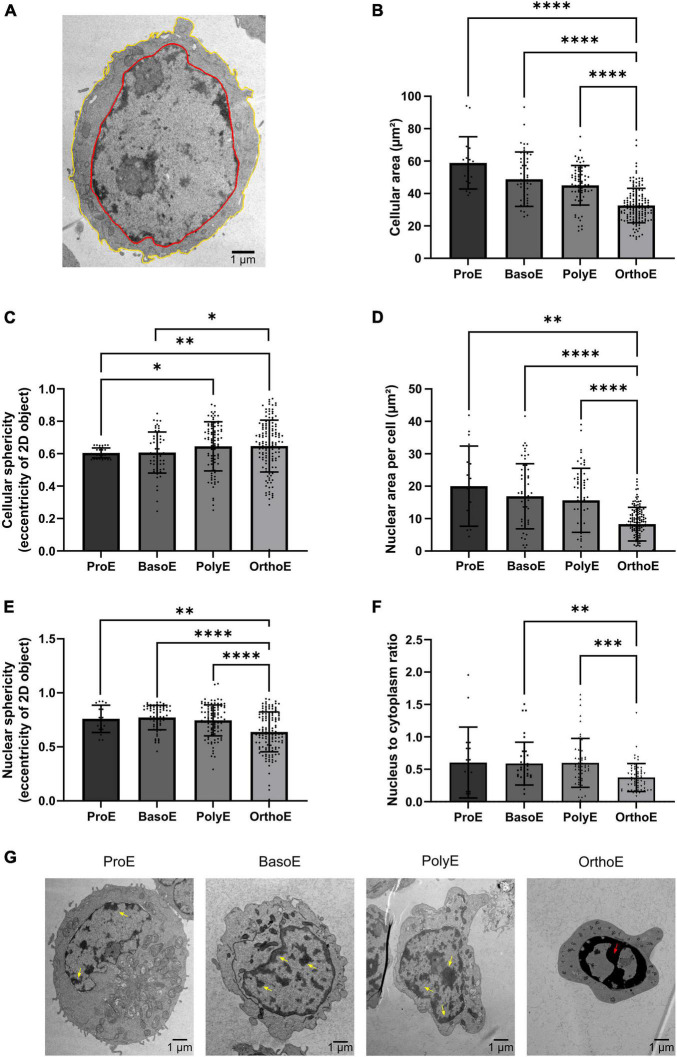
Cellular and nuclear characteristics of erythroblasts. **(A)** 3DMOD-based drawing of cellular (yellow) and nucleus (red) rims on a ProE permitting area quantification from 2D section. **(B,C)** Quantification of cellular area **(B)** and eccentricity **(C)** of erythroblasts. **(D–F)** Quantification of area **(D)**, eccentricity **(E)** of nucleus, and ratio of nucleus to cytoplasm area **(F)** in each of the differentiation stage. **(G)** Representative images of chromatin condensation from ProE to OrthoE. Yellow arrow: condensed chromatin; red arrow: pyknotic nucleus. Kruskal–Wallis analysis on at least 20 images for the three replicates. *p < 0.05; ^**^p < 0.01; ^***^p < 0.005, ^****^p < 0.001.

Electron micrographs of each stage of the terminal differentiation from ProE (Supplementary Figure 1), to BasoE (Supplementary Figure 2), to PolyE (Supplementary Figure 3), and OrthoE (Supplementary Figure 4) were analyzed. Over differentiation, erythroblast size decreases. We can follow and quantify this change by measuring the 2D area of electron microscopy sections (Figure 2A). The quantification of the average cellular area per stage shows a decrease through stages (Figure 2B). The cellular area represents 80 ± 11% for a BasoE, 77 ± 4% for a PolyE, and 61 ± 2% for a OrthoE of the cellular area of a ProE. Using the eccentricity value from the IMOD software, we can monitor the cellular roundness. Values vary from 0 (round) to 1 (elongated shape). ProE and BasoE are relatively round cells with an eccentricity value of 0.604 ± 0.007 and 0.608 ± 0.016, respectively (Figure 2C). Eccentricity value for PolyE and OrthoE is slightly higher (0.628 ± 0.029 and 0.629 ± 0.028, respectively), sign of an elongation throughout the differentiation (Figure 2C).

Nucleus size, chromatin localization, and condensation reflect the transcriptional status of the cell (Koury et al., 1988). Histological analysis describes ProE as characterized by high nuclear to cytoplasmic ratio, loose chromatin, 1 or 2 nucleoli and basophilic cytoplasm (Wickramasinghe et al., 1968, 2011). BasoE are described to have high nuclear to cytoplasmic ratio and basophilic cytoplasm but condensed chromatin and no nucleoli, whereas PolyE have round nucleus, condensed chromatin, no nucleoli, and less basophilic cytoplasm due to hemoglobin synthesis. This is the last stage to divide. OrthoE have dense pyknotic nucleus with acidophilic cytoplasm (Wickramasinghe et al., 1968, 2011). Here, we showed a decrease in nucleus size over stage (Figure 2D). Nucleus area decreases from 82 ± 10% for BasoE, 78 ± 9% for PolyE, to 42 ± 3% for OrthoE compared to ProE. We observed an increase in nucleus sphericity in OrthoE (0.63 ± 0.18 in OrthoE compared to 0.76 ± 0.12 in ProE, 0.77 ± 0.11 in BasoE, 0.74 ± 0.14 in PolyE, Figure 2E). The nucleus to cytoplasm ratio is also statistically reduced of 78 ± 8% in OrthoE compared to earlier stages (Figure 2F). Loose vs. condensed chromatin and pyknotic nucleus with high electron dense chromatin are also observed in erythroblasts (Figure 2G).

### Mitochondria Characterization Over Erythroid Differentiation

**FIGURE 3:**
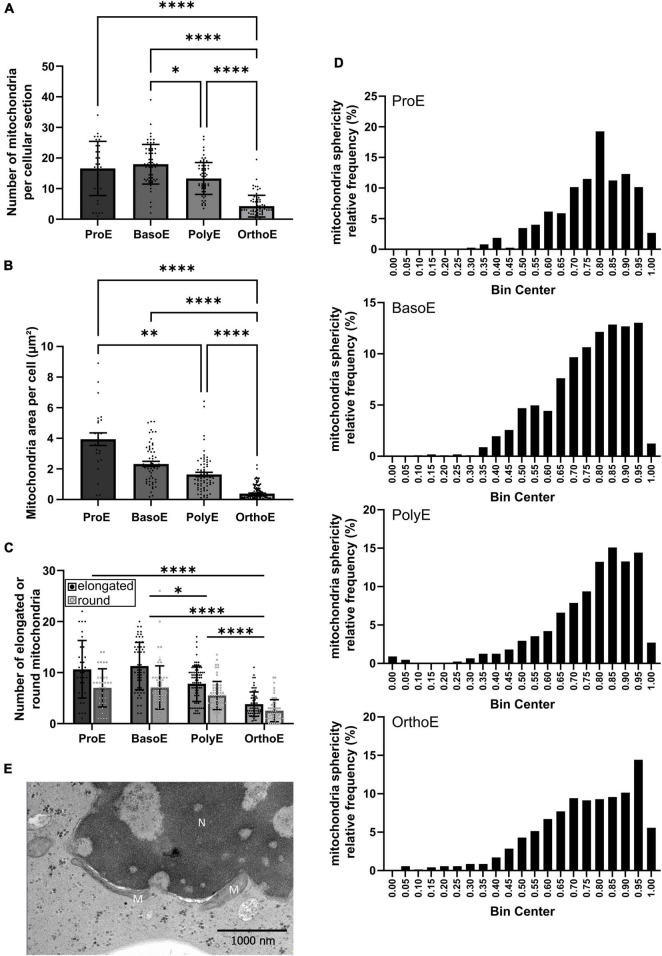
Mitochondria characterization during erythroid differentiation. **(A,B)** The number **(A)** and the total area **(B)** of mitochondria per cell were quantified in the different erythroid differentiation stages. **(C,D)** Mitochondrial morphology (elongated or rounded shape) was measured manually **(C)** or using the eccentricity quantification **(D)**. **(E)** Elongated mitochondria close to the nucleus of an OrthoE. M, mitochondria; N, nucleus. Kruskal–Wallis analysis on at least 20 images for the three replicates. *p < 0.05; ^**^p < 0.01; ^****^p < 0.001.

By flow cytometry and biochemical approaches, we have previously showed progressive clearance of mitochondria starting from the transition between BasoE and PolyE (Moras et al., 2020, 2022). Here, we followed mitochondria quantity per cell. We observed a decreased in mitochondria number starting from BasoE (17.98 ± 0.810 mitochondria sections per cell) to PolyE (13.30 ± 0.631 mitochondria sections per cell) transition, with a subsequent decrease observed in OrthoE (4.28 ± 0.429 mitochondria sections per cell) (Figure 3A). This phenomenon is also detected when analyzing the total area of mitochondria sections per cell between erythroblast stages (Figure 3B).

Changes in mitochondrial metabolism, oxidative stress, or regulation of mitochondria abundance within cells can be accompanied by changes in mitochondrial morphology. Mitochondrial functions are tightly regulated over erythroid differentiation (Oburoglu et al., 2014, 2016; Gonzalez-Menendez et al., 2021; Rossmann et al., 2021). Mitochondria fragmentation is an essential step for maturation (Gonzalez-Ibanez et al., 2020). Rounded and elongated mitochondria can be observed in all differentiation stages (Supplementary Figure 5). Here, we analyzed mitochondrial morphology first manually (Figure 3C) and second using the eccentricity quantification (Figure 3D). We observed a progressive change in mitochondria shape from elongated to more rounded shape within the differentiation stages (Figure 3C). Looking at the distribution of mitochondria eccentricity values, OrthoE exhibit a left shift toward more rounded forms (OrthoE vs. PolyE, *p* = 0.0026) (Figure 3D). In OrthoE interestingly, the mitochondrial morphology is not homogeneous, and some of the most elongated mitochondria were observed, always in close proximity to the nucleus (Figure 3E).

**FIGURE 4:**
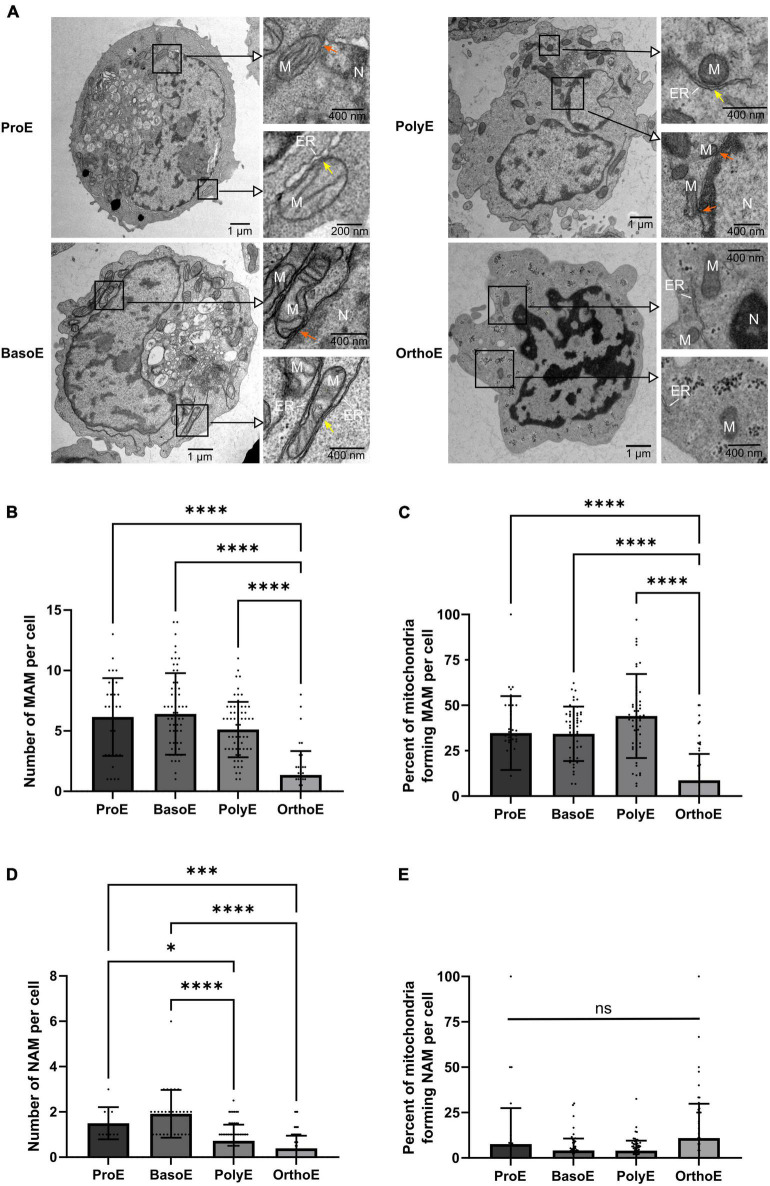
Quantification of mitochondria-organelle contact sites. **(A)** Representative images of erythroblasts at various stages with insets on mitochondria. Orange arrow: mitochondria-ER contact site, yellow arrow: mitochondria-nucleus contact site. M, mitochondria; N, nucleus; ER, endoplasmic reticulum. **(B)** Quantification of mitochondria-endoplasmic reticulum (MAM) per cell at each stage. **(C)** Percentage of mitochondria forming MAM contact site per cell. **(D)** Quantification of nucleus-mitochondria (NAM) per cell at each stage. **(E)** Percentage of mitochondria forming NAM contact site per cell. Kruskal–Wallis analysis on at least 20 images for the three replicates. *p < 0.05; ****p < 0.001.

Mitochondria can form contact sites with virtually all organelles in the cell (Lackner, 2019). These structures are involved in protein and lipid transport, calcium homeostasis, vesicular trafficking, mitochondrial dynamic, apoptosis, and autophagy (Ellenrieder et al., 2017; Giamogante et al., 2020). Regardless of organelle and cristae morphology, mitochondrial functionality can be studied through the quantification of interorganelle contact sites, as for example mitochondria-associated membranes (MAMs) adapt to glucose availability to regulate mitochondrial dynamics and bioenergetics (Theurey et al., 2016). Such contact sites can be observed in human erythroblasts (Figure 4A). Membrane–membrane contact of several organelles is determined by specific protein complex and has an intermembrane distance of approximately 5–8 nm (Zanetti and Mayorga, 2009). This is the maximal distance measured between both membranes, which we have considered as a positive membrane–membrane contact site. The number of MAM, which bring together mitochondria and endoplasmic reticulum membranes, is relatively constant from ProE to PolyE (6.15 ± 3.22 in ProE, 6.40 ± 3.38 in BasoE, 5.12 ± 2.29 in PolyE, Figure 4B). Concomitant with the decrease in mitochondria abundance in this stage, the number of MAMs decreases significantly in OrthoE (1.35 ± 1.97, Figure 4B). On mitochondria-containing erythroblasts, we analyzed the percentage of mitochondria forming MAMs in different erythroblast stages. Despite the decrease in mitochondria number, we observe 34.73 ± 20.32% in ProE, 34.27 ± 15.02% in BasoE, 44.10 ± 23.10% in PolyE, and only 8.687 ± 14.49% in OrthoE of mitochondria forming MAMs (Figure 4C).

Mitochondria are also known to make contact with the nuclear membrane, forming nucleus-associated mitochondria (NAM; Desai et al., 2020; Eisenberg-Bord et al., 2021). As a part of the retrograde signaling response that links mitochondria homeostasis to the activation of adaptative response from transcriptional control within the nucleus, NAM participate in the regulation of many cellular processes such as cell proliferation, stress adaptation, metabolic switch, autophagy, and mitochondrial biogenesis (Strobbe et al., 2021). We quantified NAM in erythroblasts based on the same criteria of distance as previously described for MAMs (Zanetti and Mayorga, 2009). Whereas this structure appears to be relatively rare with a mean of 1.50 ± 0.22 in ProE and 1.92 ± 0.19 in BasoE, they are statistically decreased in Poly (0.72 ± 0.09) and OrthoE (0.39 ± 0.07) (Figure 4D). The percentage of mitochondria forming NAM remain unchanged between differentiation stages (Figure 4E). This might suggest a specific removal of a subset of mitochondrial population, maintaining those in proximity to the nucleus despite the total reduced number of this structure (Figure 3E).

### Cellular Trafficking and Autophagy

**FIGURE 5:**
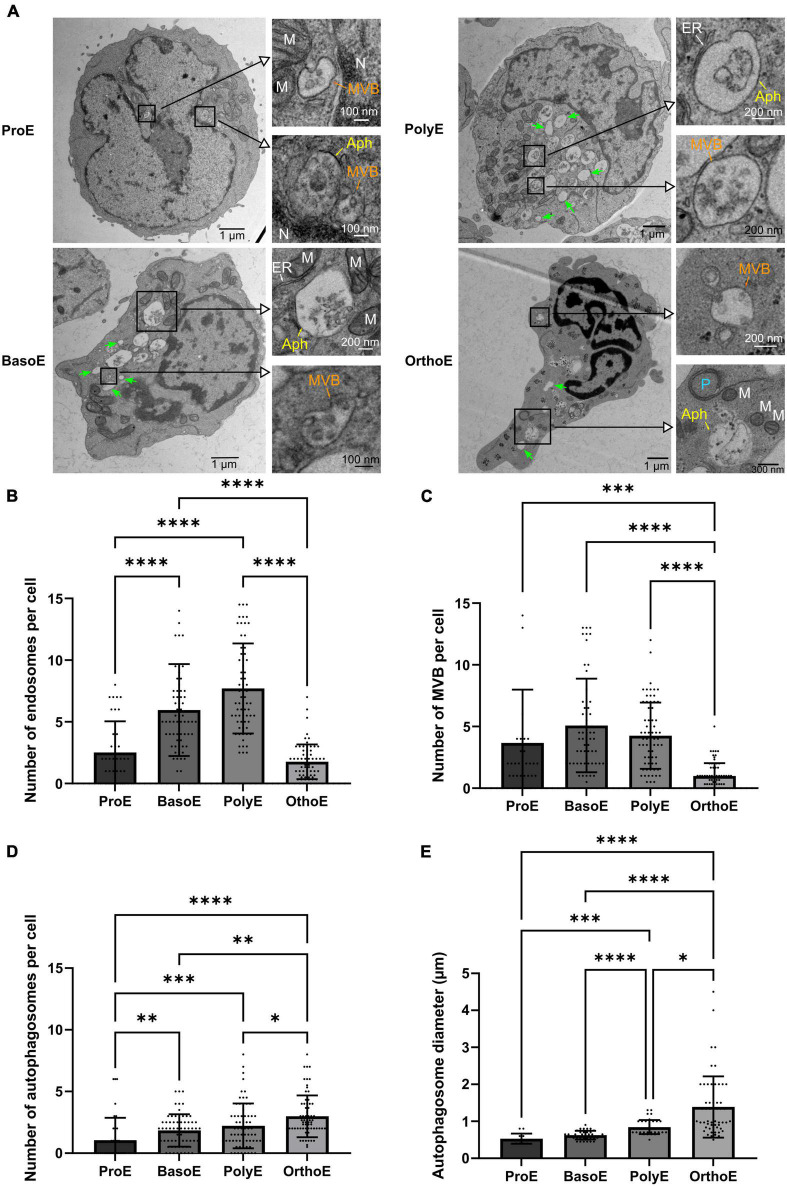
Characterization of the endosomal and autophagic pathway during erythroid differentiation. **(A)** Representative images of erythroblasts at various stages with insets on intracellular vesicles. Green arrow: endosomes, MVB, multivesicular body; Aph, autophagosome; P, phagophore; M, mitochondria; N, nucleus; ER, endoplasmic reticulum. **(B–D)** Quantification of endosomes **(B)**, multivesicular bodies **(C)**, and autophagic vesicles **(D)** in each differentiation stage. **(E)** Measure of the diameter of the autophagic structures over the different erythroid differentiation stages. Kruskal–Wallis analysis on at least 20 images for the three replicates. *p < 0.05; **p < 0.01; ***p < 0.005, ****p < 0.001.

Endosomes and multivesicular bodies (Figure 5A) are both structures involved in iron uptake, and also transferrin and transferrin receptor recycling, the first steps in the heme biosynthesis pathway (Killisch et al., 1992). BasoE and PolyE presented higher number of these structures with a mean of 5.96 ± 3.72 endosomes per BasoE and 7.7 ± 3.36 endosomes per PolyE (Figures 5B,C). On the contrary, the mean number of endosomes in OrthoE drastically drops to 1.7 ± 1.4 (Figure 5B). The pattern of MVB abundance is similar with one of the endosomes as with MVBs that are more represented in BasoE and PolyE (mean of 5.09 ± 3.7 structures per cell in BasoE and 4.26 ± 2.69 in PolyE), whereas OrthoE contain only 1.04 ± 0.98 MVBs per cell (Figure 5C).

Through terminal erythropoiesis, cell size decreases and organelles are progressively removed. Several evidences point out the role of general autophagy and/or specific mitophagy in terminal mitochondrial clearance (Schweers et al., 2007; Kundu et al., 2008; Zhang et al., 2009; Betin et al., 2013; Honda et al., 2014; Moras et al., 2022). Here, we showed a progressive increase in autophagosome number with a mean of 1.05 in ProE, 1.84 in BasoE, 2.21 in PolyE, and 2.99 autophagosomes per cell in OrthoE (Figure 5D). Concomitantly, the diameter of autophagosomes that reflect the size of the structure in a 2S section is also progressively increasing through the differentiation (0.53 μm in ProE, 0.63 μm in BasoE, 0.84 μm in PolyE, and 1.39 μm in OrthoE, Figure 5E). This is the sign of an intense activation of autophagic pathways in late stages of erythropoiesis.

### Glycogen Accumulation

**FIGURE 6:**
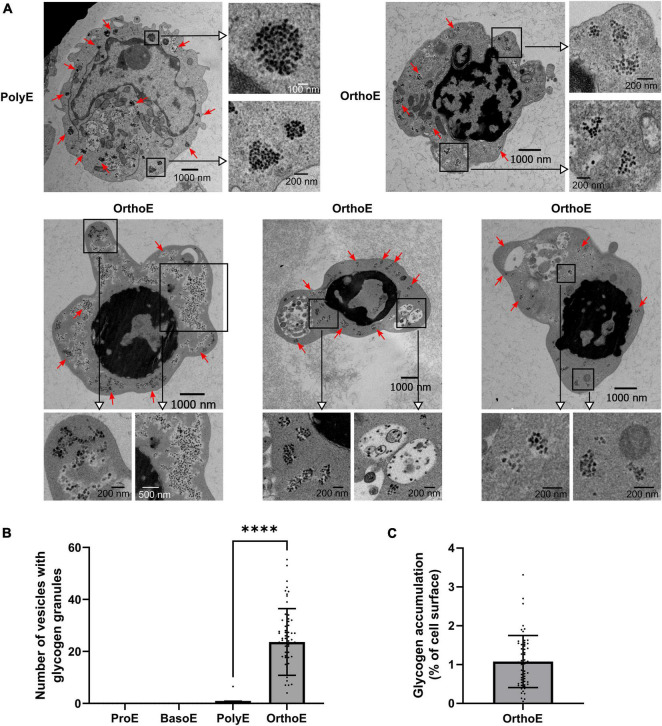
Glycogen accumulation in OrthoE. **(A)** Representative images of the only ProE exhibiting glycogen granules and several OrthoE with inset on glycogen granules (red arrows). **(B)** Quantification of vesicles containing glycogen granules and **(C)** percentage of cell surface occupied by glycogen granules (surface of glycogen granules relative to cell surface × 100) in OrthoE. Kruskal–Wallis analysis on at least 20 images for the three replicates. ^****^p < 0.001.

In early expansion of erythropoiesis – from hematopoietic stem cell (HSC) to colony-forming unit-erythroid (CFU-E) – cell metabolism switches from glycolytic and proliferative to oxidative and differentiating metabolism (Oburoglu et al., 2014; Bonora et al., 2015; Schell and Rutter, 2017). In late stages, we could observe dark granules that we identified as glycogen granules, according to the literature (Revel et al., 1960; Castejon et al., 2002; Ørtenblad et al., 2013; Vezzoli et al., 2020; Figure 6A). Except in one PolyE cell out of 106 images, glycogen granules were found only in OrthoE (Figure 6A). We quantified up to 65 vesicles per cell (Figure 6B) that represent around 1% of the cell surface (Figure 6C) in OrthoE, the stage when most of mitochondria were lost (Figure 6B).

## Discussion

Well-admitted morphological changes regarding cell and nucleus morphology observed throughout human erythroid are detectable on electron microscopy pictures. From 2D cellular sections, we quantified the reduction in cell and nucleus size and roundness (eccentricity). In histology books, proerythroblast size is 14–19 μm, basophilic erythroblast size is 12–17 μm, polychromatophilic erythroblast size is 12–15 μm, and orthochromatic erythroblast size is 8–12 μm. According to these data, the reduction in size from ProE to OrthoE is 60%. Only differences with OrthoE are significant in our analyses of TEM 2D sections but the decrease percentage of each stage is reproduced. Finally, we were also able to observe the progressive chromatin condensation (Figure 2).

Focusing on mitochondria, we showed its progressive clearance along the terminal maturation with a significant switch between PolyE and OrthoE (Figure 3). We also observed a shift toward more rounded shape along the differentiation (Figure 3). Mitochondria are highly dynamic organelles; therefore, the number of mitochondria reflects the overall aspect of the mitochondrial network. Gonzalez-Ibanez described recently the importance of FIS1 and MFN1 in the control of mitochondria shape during erythropoiesis, showing that mitochondria fragmentation is a prerequisite for proper progression through differentiation (Gonzalez-Ibanez et al., 2020). In relation to its dynamic, mitochondria are important organelles reflecting the energetic status of the cell (Benard et al., 2007). Under conditions of high energetic demand, mitochondria are abundant, fused, and provide the cells with high levels of ATP. Erythroblasts were described to undergo metabolic switch to synthetize specialized proteins allowing the mature red blood cell to be fully functional (Oburoglu et al., 2016). This is in agreement with the elongated shape of mitochondria that we observed at ProE stage and the progressively more rounded morphology we described throughout terminal erythropoiesis.

Functions of mitochondria are tightly linked to communication among organelles (Theurey et al., 2016; Desai et al., 2020; Gao et al., 2020; Kohler et al., 2020; Yang et al., 2020; Strobbe et al., 2021). The quantification of MAMs shows no difference from ProE to PolyE. At the transition from PolyE to OrthoE, the total of MAM structures per cell decreases together with the percentage of mitochondria forming MAMs (Figure 4). MAMs are well-known structures involved in regulation of calcium homeostasis, lipid synthesis and transfer, mitochondria movement and dynamics, ER stress regulation, inflammation, and autophagy (Gao et al., 2020; Kohler et al., 2020; Yang et al., 2020). OrthoE stage also corresponds to a stage with drastic changes in cellular functions, where the cell needs to shut down several functions and to prepare for enucleation. On the one hand, lacking contact points between organelles could simply result from the decrease in organelles abundance. Nevertheless, our observations suggest that there is a specific decrease of MAM structures not related to the overall mitochondria clearance that can be related to the loss of relevance of MAM-associated structures over differentiation. This speculation is supported by our recent findings which demonstrate that mitochondrial clearance and enucleation were strongly diminished when VDAC1 and TSPO1 were downregulated (Moras et al., 2020, 2022). Indeed, these proteins are mitochondrial outer membrane proteins known to be involved in outer MAM contact sites and also heme and cholesterol traffic.

Regarding mitochondria or nucleus communication, we showed that the percentage of mitochondria forming NAM is not different among stages, whereas the total amount of NAM decreases in PolyE and OrthoE compared to ProE and BasoE. This might suggest a specific removal of a subset of mitochondrial population, maintaining those in proximity to the nucleus despite the total reduced number of these structures. NAM were first described 50 years ago (McCully and Robinow, 1971; Franke et al., 1973) and they were speculated to participate in several functions as yeast fission and heme and phospholipid traffic in yeast (McCully and Robinow, 1971; Franke et al., 1973; Martinez-Guzman et al., 2020; Eisenberg-Bord et al., 2021). They are described to regulate the localization of transcription factors and consequently controls cellular adaptation to stress by retro-communicating with the nucleus (Jazwinski, 2014; Desai et al., 2020; Strobbe et al., 2021). Precise regulation of mitochondrial activity is essential to complete an efficient enucleation (Gonzalez-Menendez et al., 2021; Liang et al., 2021). In fact, perinuclear localization of functional mitochondria is required for mice enucleation (Liang et al., 2021). Here, we showed that whereas some mitochondria appear small and rounded in OrthoE, some were elongated and in close proximity to the nucleus (Figure 3E). This might suggest that not all mitochondria in OrthoE are committed to the same function. Subcellular repartition of mitochondria with different morphologies and specialized functions has been identified in muscles (Willingham et al., 2021). The same might be true for OrthoE and should be taking into account while functionality studies might be accounted for different subpopulations of mitochondria within the cell.

Another role of mitochondria is to synthetize heme, a crucial component of hemoproteins as hemoglobin (Dailey and Meissner, 2013; Hamdi et al., 2016). In erythroid cells, hemoglobin synthesis starts from BasoE to PolyE transition and is accompanied by a strong intracellular trafficking (Rio et al., 2019). We highlighted in Figure 5 an increase in endosomes number from ProE to PolyE. This can be the reflection of the stimulation of the intracellular trafficking to import iron and heme, as expected for differentiation stages presenting the higher rate of hemoglobin synthesis. PolyE is also the pivotal stage where erythroid membrane proteins are translated and addressed to the plasma membrane (Blanc et al., 2005; Gautier et al., 2016). This process involved trafficking from the ER to the plasma membrane and would participate to the high level of intracellular trafficking that is observed at this maturation stage. PolyE are also the latest stage to divide. In OrthoE, transcription is highly reduced and also protein synthesis (Ludwig et al., 2019). This is consistent with the lower activity of the endocytosis or recycling pathway we observed (Figures 5B,C). MVBs, also known as exosomes, are reduced at OrthoE stage. Whereas it is known that MVBs are involved in exocytosis in an erythroid context (Fader and Colombo, 2006), this pathway seems not to be the major way of unneeded cellular components clearance.

Mammals’ terminal erythropoiesis requires the clearance of organelles and the enucleation to achieve final mature erythrocyte. These processes occur at OrthoE and reticulocyte stages, where the activation of general autophagy and/or specific mitophagy occurs (Schweers et al., 2007; Kundu et al., 2008; Zhang et al., 2009; Betin et al., 2013; Honda et al., 2014; Moras et al., 2022). Supporting these reports, we observed here an increase in autophagosome abundance and size in OrthoE (Figure 5) that might reflect an increase in autophagic activity within the cell. Defect in proper mitochondria degradation leads to the maintenance of mitochondria in mature circulating red blood cells, as recently described for SCD or for SLE (Jagadeeswaran et al., 2017; Caielli et al., 2021; Martino et al., 2021). While not completely elucidated, this defective mitochondrial clearance might participate in the pathophysiology of the diseases.

Adaptative changes in OrthoE metabolism could also be highlighted by the presence of glycogen granules (Figure 6). Energy requirement is low in OrthoE (Gonzalez-Menendez et al., 2021). Within the cell, allosteric regulation of glycolytic enzymes prevents glucose to be processed by glycolysis and divert the flux toward storage forms. Without knowing whether this accumulation is a side effect of *ex vivo* differentiation, accumulation of glycogen can be indicating of a preparatory mechanism to provide enucleated red cells with glycolysis substrates. In mature red blood cells devoid of functional mitochondria, the energetic metabolism relies on glycolysis. Moreover, glucose also fueled the pentose phosphate pathway, leading to the production of NADPH, the major coenzyme required to replenish glutathione-based antioxidative defenses. To better understand this phenomenon, further experiments will be required to confirm the glycogen granules accumulation *in vivo* and to study the molecular players involved in this pathway.

**FIGURE 7:**
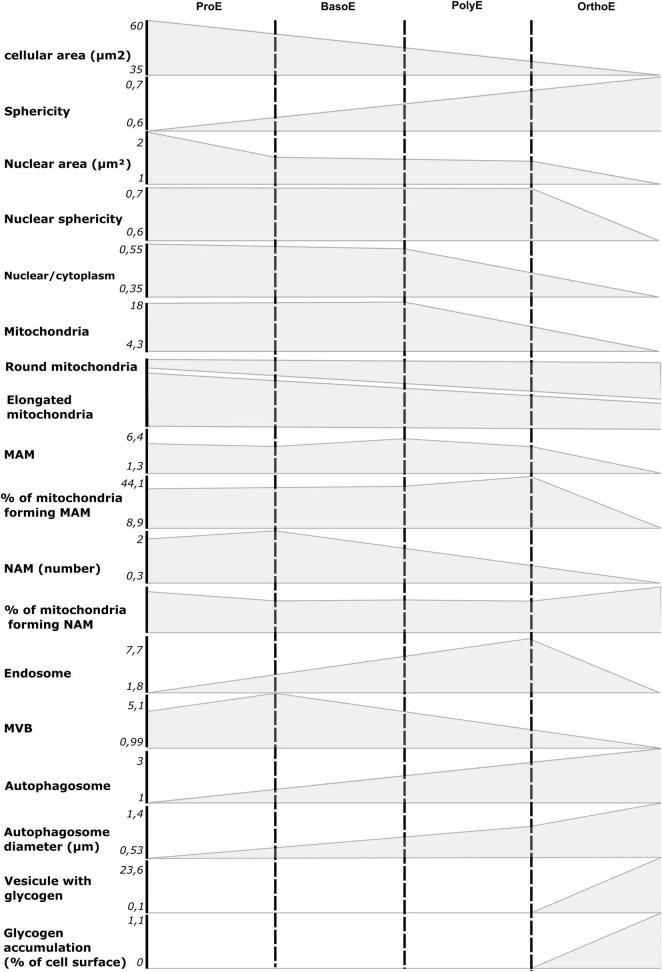
Summary of morphological and ultrastructural quantifications from our analysis. The mean value for ProE to OrthoE stages was reported on ordinate for each quantified item presented in this article.

In summary, we showed that TEM is a valuable tool to study morphological and ultrastructural changes within erythroblasts (Figure 7). We showed the decrease in size of the cell and the nucleus and also the chromatin condensation, hallmark of the transcription arrest throughout terminal erythropoiesis. Insights into organelles morphology and cellular metabolism can also come from micrographs. In this regard, we could monitor the progressive decrease in mitochondria abundance, the mitochondrial fragmentation, the decrease in interorganelle communication by the reduction in MAM and NAM count, the elevated intracellular trafficking from the endosomal pathway, and the induction of autophagy. All of these processes are essential for proper erythropoiesis, and we believe that characterization of ultrastructural changes in erythroblasts would be of a great interest to further investigate the origin of dyserythropoietic diseases.

## Data Availability Statement

The original contributions presented in the study are included in the article/Supplementary Material, further inquiries can be directed to the corresponding author.

## Ethics Statement

This study was approved and conducted according to the Institutional Ethical Guidelines of the National Institute of Blood Transfusion (N°2019-1, INTS, Paris, France). The patients/participants provided their written informed consent to participate in this study.

## Author Contributions

MO and SL conceived the project and obtained funding. SL designed the experiments. AD, MM, and SL performed the experiments. JL evaluated protocols and supervised blood samples purchasing. AD, JJ, CS, J-MV, CF, and SL analyzed the data. MO, CF, J-MV, and SL critically evaluated the experiments. AD, CF, MO, and SL wrote the manuscript. MO, CF, and SL provided supervision. All authors read and commented on the manuscript and approved the final version.

## Conflict of Interest

The authors declare that the research was conducted in the absence of any commercial or financial relationships that could be construed as a potential conflict of interest.

## Publisher’s Note

All claims expressed in this article are solely those of the authors and do not necessarily represent those of their affiliated organizations, or those of the publisher, the editors and the reviewers. Any product that may be evaluated in this article, or claim that may be made by its manufacturer, is not guaranteed or endorsed by the publisher.

## Supplementary Material

Supplementary Figure 1 Electron micrographs of ProE.

Supplementary Figure 2 Electron micrographs of BasoE.

Supplementary Figure 3 Electron micrographs of PolyE.

Supplementary Figure 4 Electron micrographs of OrthoE.

Supplementary Figure 5 Mitochondrial morphology in erythroblasts. Representative images of rounded and elongated mitochondria in erythroblasts with inserts of rounded (R) or elongated mitochondria (E).
