# Supplementary figures and images for: Transmission Electron Microscopy to Follow Ultrastructural Modifications of Erythroblasts Upon ex vivo Human Erythropoiesis
Source: Front Physiol. 2022 Feb 9;12:791691. doi: 10.3389/fphys.2021.791691 (PMC8864112; doi:10.3389/fphys.2021.791691)

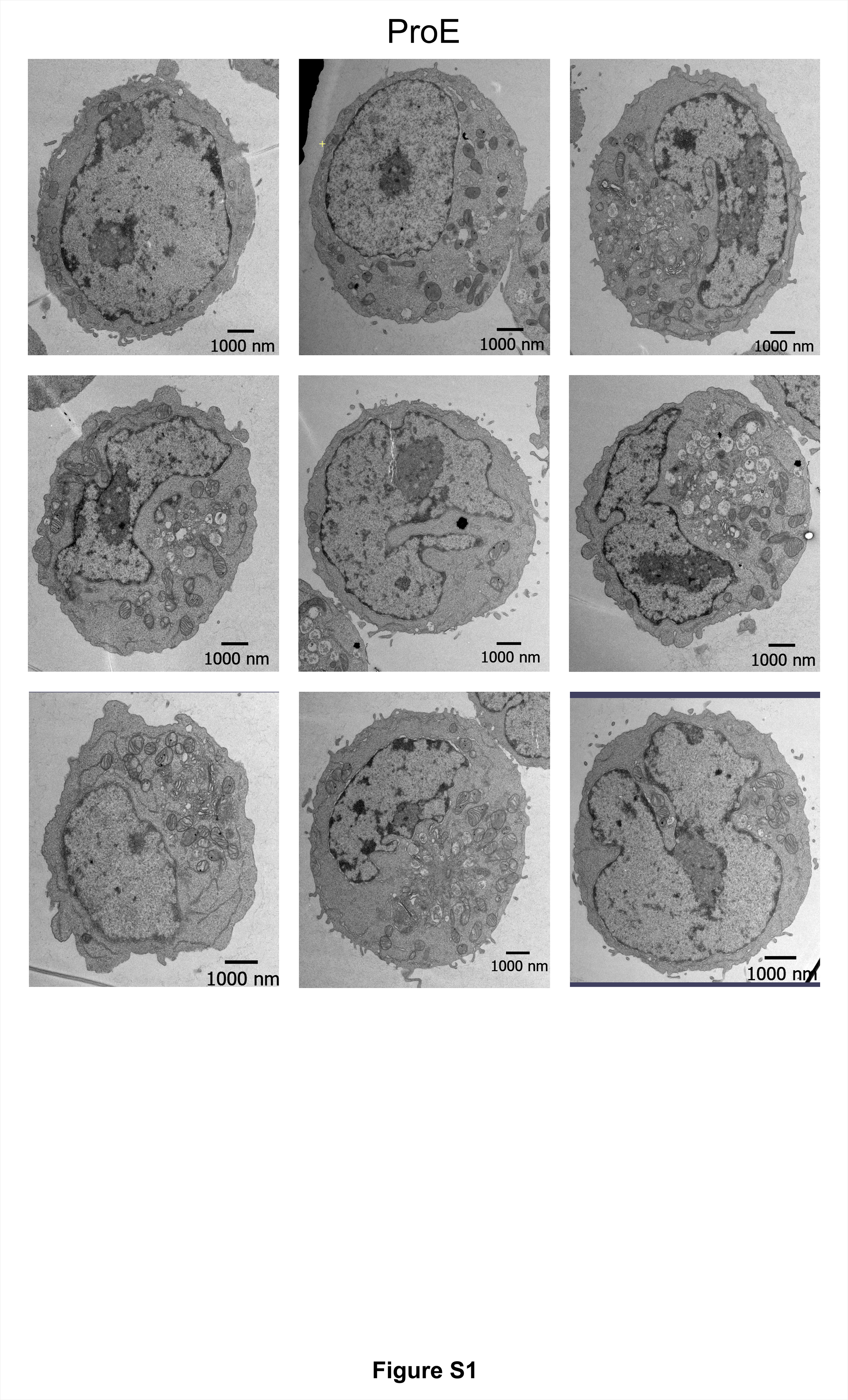

Supplement: Supplementary Figure 1 — Electron micrographs of ProE. [file Image_1.TIF]

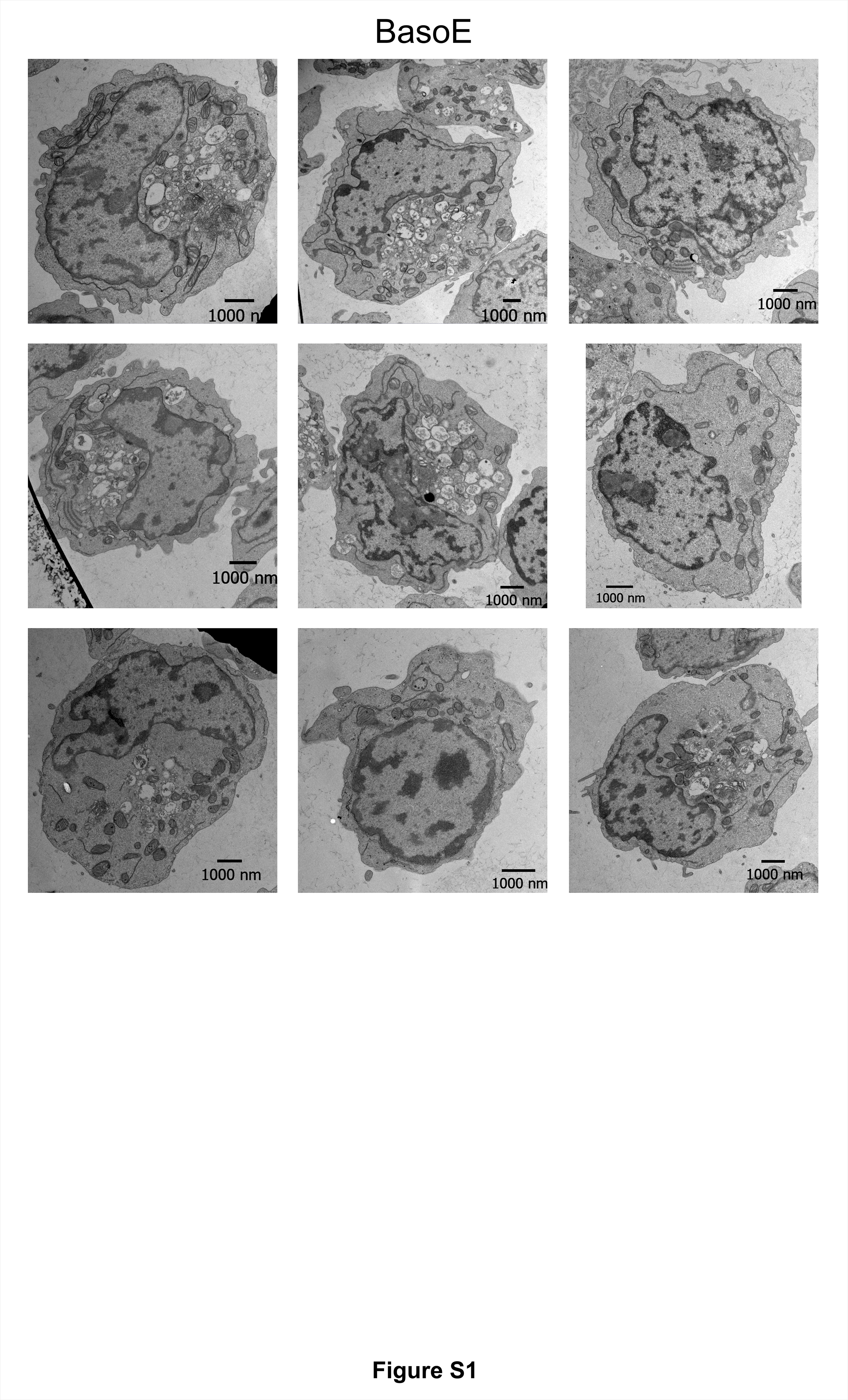

Supplement: Supplementary Figure 2 — Electron micrographs of BasoE. [file Image_2.TIF]

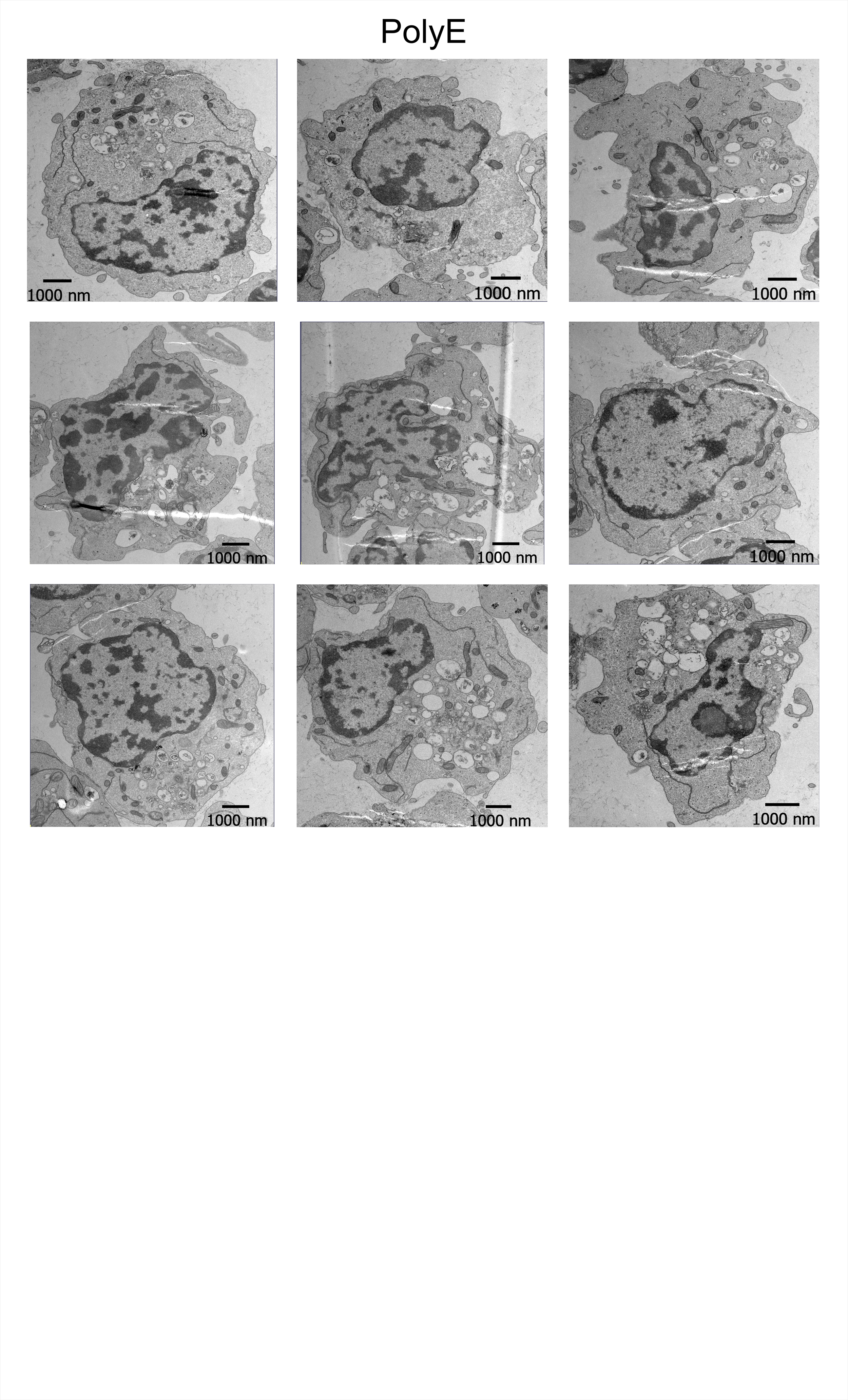

Supplement: Supplementary Figure 3 — Electron micrographs of PolyE. [file Image_3.TIF]

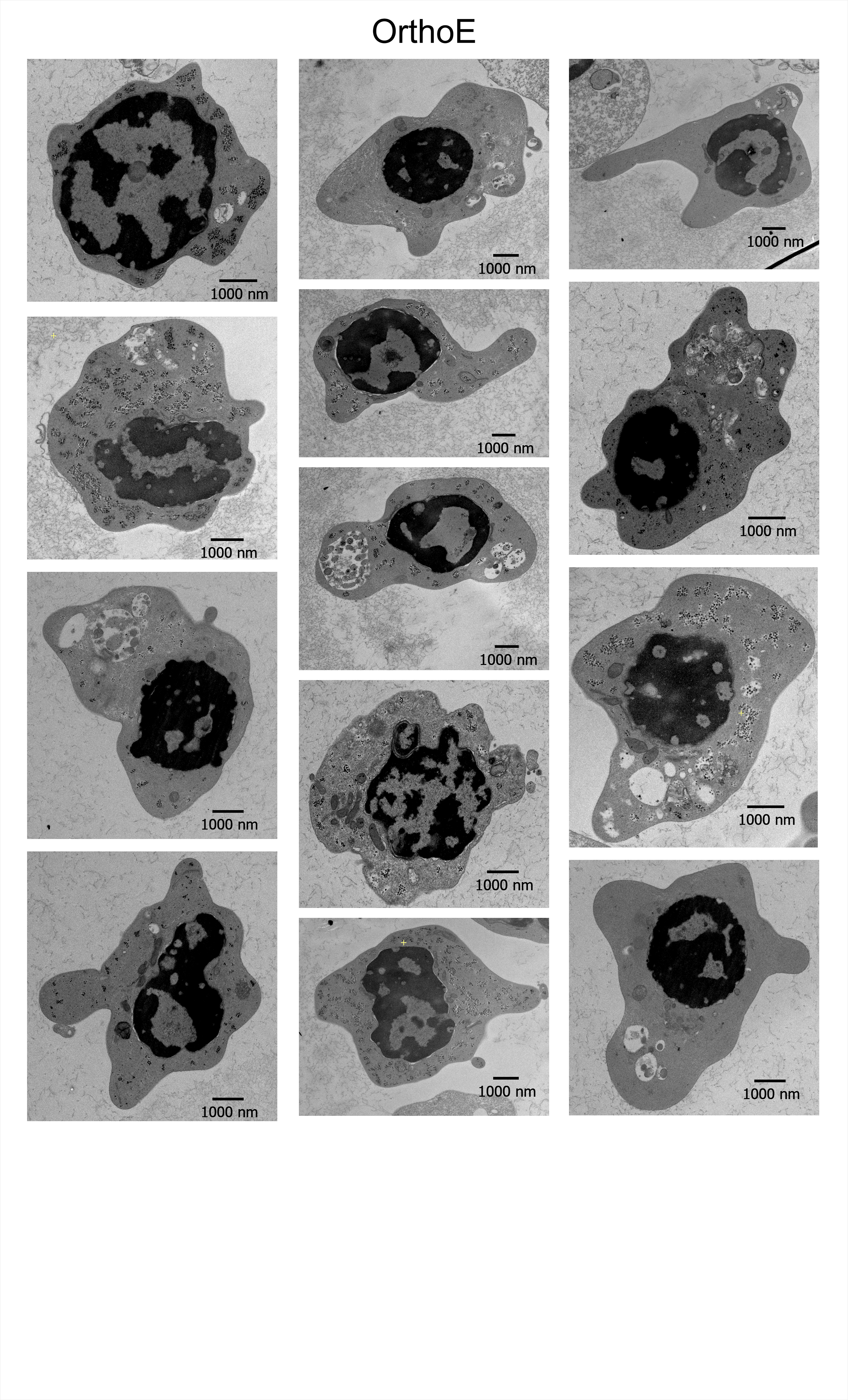

Supplement: Supplementary Figure 4 — Electron micrographs of OrthoE. [file Image_4.TIF]

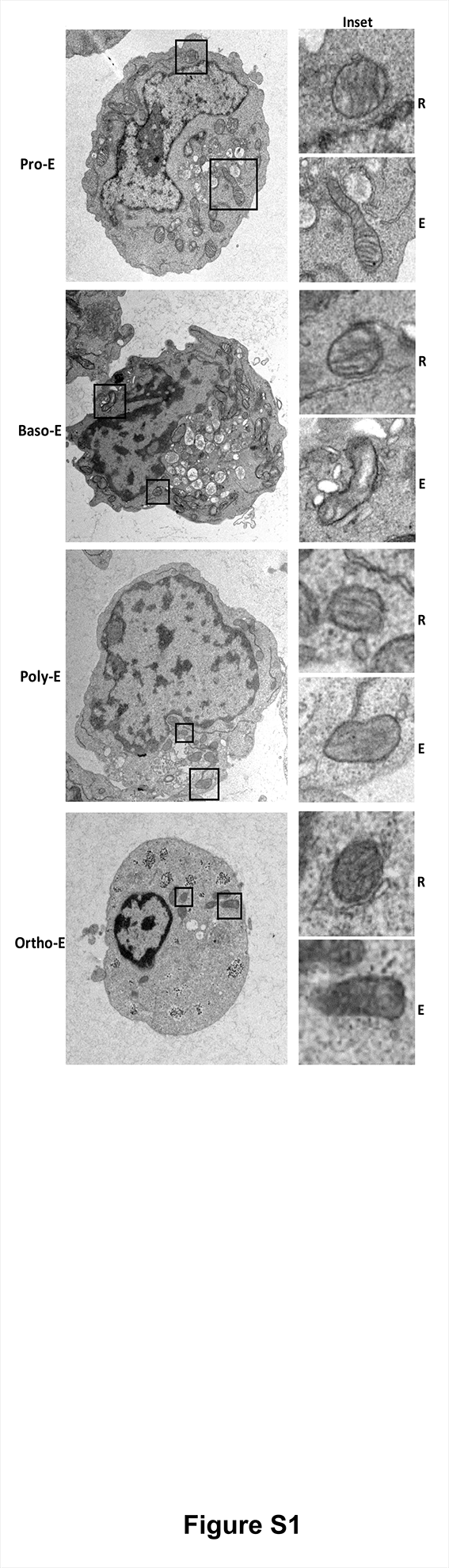

Supplement: Supplementary Figure 5 — Mitochondrial morphology in erythroblasts. Representative images of rounded and elongated mitochondria in erythroblasts with inserts of rounded (R) or elongated mitochondria (E). [file Image_5.TIFF]
